# Non-invasive preoperative MRI-based deep transfer learning radiomics model for predicting meningioma brain invasion

**DOI:** 10.3389/fonc.2026.1935311

**Published:** 2026-10-07

**Authors:** Dong Yuan, Jiajia Zhang, Chao Zhang, Xiyue Jing, Quanzhi Feng, Tong Han

**Affiliations:** 1 Department of Radiology, Huanhu Hospital Affiliated to Tianjin Medical University, Tianjin, China; 2 Tianjin Key Laboratory of Cerebral Blood Flow Reconstruction and Head and Neck Tumor New Technology Translation, Tianjin, China; 3 Department of Radiology, Nayong Xinli Hospital, Bijie, Guizhou, China; 4 Institute of Neurosurgery, Huanhu Hospital Affiliated to Tianjin Medical University, Tianjin, China

**Keywords:** 2.5D imaging, brain invasion, deep transfer learning, deep transfer learning radiomics, meningioma

## Abstract

**Background:**

Current preoperative assessment of meningioma brain invasion (BI) demands imaging analytical frameworks that optimize computational efficiency while capturing multidimensional tumor-pathology correlations. Building upon the inherent advantages of 2.5D MRI in synthesizing multiplanar biological data with manageable processing loads, this study innovatively integrates deep transfer learning (DTL), a technique bridging pretrained network knowledge with target domain adaptation, to systematically identify tumor-invasive biological signatures and develop an optimized predictive system using preoperative contrast-enhanced T1-weighted imaging (CE-T1WI).

**Methods:**

This retrospective study enrolled 674 patients with pathologically confirmed meningiomas, including 53 with BI and 621 without BI. The cohort was stratified into a training set and an independent test set. Tumor regions of interest (ROIs) were delineated on CE-T1WI, and radiomics and DTL features were extracted. Radiomics and DTL features were fused via early fusion, followed by feature selection. Within the training cohort, five-fold cross-validation was performed, with SMOTE applied exclusively to the training portion of each fold; validation folds and the independent test set retained their original class distributions.

**Results:**

The effectiveness of the models was compared using the area under the ROC curve (AUC). In the training set, ComM achieved an AUC of 0.977, while in the test set, its AUC reached 0.935. ComM demonstrated the best performance (training set: AUCComM > AUCDTLRM > AUCRadM > AUCClinM > AUCDTLM; test set: AUCComM > AUCDTLRM > AUCRadM > AUCDTLM > AUCClinM), showing excellent preoperative predictive capability for meningioma BI. The Hosmer-Lemeshow test indicated good model fit, and the calibration curve revealed that ComM was closest to the ideal curve.

**Conclusion:**

ClinM, RadM, DTLM, DTLRM, and ComM based on 2.5D CE-T1WI all exhibited good performance in predicting meningioma BI. The clinical-radiomics-DTL fusion model (ComM) demonstrated the highest efficacy, suggesting that DTL provides additional features correlated with meningioma BI that differ from radiomics features. This model shows potential clinical utility for preoperative prediction; however, further external validation in larger multicenter cohorts is required before clinical implementation.

## 1 Introduction

Meningiomas are the most common primary intracranial tumors. Brain invasion (BI) has been added as a grading criterion for Grade II meningioma in the new 2016 World Health Organization (WHO) classification (1), which has a direct impact on the diagnosis and grading of meningiomas, therapeutic decision-making, and prognostic prediction. Currently, pathology is the gold standard for the diagnosis of BI in meningiomas; however, this approach may lead to misdiagnosis or missed diagnosis and increase the risk of recurrence due to the inability of the surgeon to accurately obtain samples, and the pathologist can only passively analyze the specimen submitted for examination.

Through radiomics, deep learning (DL) and other algorithms, machine learning can mine intrinsic hidden information in images that corresponds to the biological behaviors of BI (2). This information can be used to predict and assess BI status before surgery, which can provide clinicians with more comprehensive information about the tumor, assist in the formulation of surgical strategies and improve the diagnosis of BI, reducing the possibility of recurrence and improves patient prognosis.

The potential value of preoperative images in predicting BI of meningiomas has been gradually explored. Most studies have acknowledged that radiomics is an effective tool for identifying meningioma BI. Conventional low-order radiomics features (e.g., shape features, intensity features, texture features) are based on predefined features of tumor images, which inadequately reflect the imaging heterogeneity of tumors (3). In contrast, DL can mine extremely high-level data from a large amount of raw data for characterization (4), and the addition of DL features may improve the predictive performance of traditional radiomics models. However, current DL research still faces many practical problems, such as hardware requirements, complex model design, poor applicability, low explainability and tendency toward bias. Therefore, building a large-scale, high-quality dataset, which is required for standard DL models, is laborious and time-consuming. Many studies have used transfer learning strategies to address many of these difficulties (5). Transfer learning uses the knowledge obtained in the source domain and source task to improve the learned target predictive function for the target task in the target domain, especially when there are scarce data in the target domain and ample data in the source domain (6). Deep transfer learning (DTL) is a transfer learning method based on DL that can effectively utilize previously learned knowledge with a minimal amount of training data or model fine-tuning to solve new tasks (7).

Pan et al. (8) defined DTL as the transfer of knowledge learned from the source domain to task learning in the target domain, which improves the performance of the model in target domain task prediction; briefly, DTL is the application of previously acquired knowledge to learn new knowledge. Recent advances in medical imaging AI have extended beyond conventional convolutional neural networks. Foundation models based on large-scale pretraining have shown potential to provide transferable representations that can be adapted to a range of downstream radiological tasks (9). Vision Transformers have also attracted increasing attention because of their ability to capture long-range spatial dependencies and global contextual information in medical images (10). In parallel, multimodal AI approaches that integrate imaging information with clinical metadata or other complementary data sources are increasingly being explored to improve disease characterization and prediction (11). Explainable AI has also become an important area of research in medical imaging, aiming to improve the transparency, interpretability, and clinical trustworthiness of AI-assisted predictions (12).

Previous studies (13, 14) have mostly used the maximum slice of tumors (i.e., 2D) to extract DL features, but the information obtained within tumors is limited. The 2.5D images of tumors (i.e., the maximum slice and the slices above and below 1 mm and 2 mm) overcome the disadvantage that traditional 2D models lack surrounding 3D data and reduce the demand for training data and model parameters (15). There have been no reports on the use of DTL features combined with radiomics features for the preoperative prediction of meningioma BI. Therefore, we explored the relationship between BI and DTL and radiomics features and established a combined clinical-radiomics-DTL model to predict meningioma BI.

## 2 Methods

### 2.1 Study subjects

This retrospective study was approved by the Institutional Review Board of Tianjin Huanhu Hospital, and the requirement for informed consent was waived. A total of 1,073 patients with pathologically confirmed meningiomas who underwent preoperative MRI between September 2021 and October 2023 were retrospectively screened. The patient selection process is illustrated in **Figure 1**. After applying the inclusion and exclusion criteria, 674 consecutive patients (mean age, 57 years; range, 11–82 years; 192 men [28.5%]) were included, comprising 621 patients without BI and 53 patients with BI.

**Figure 1:**
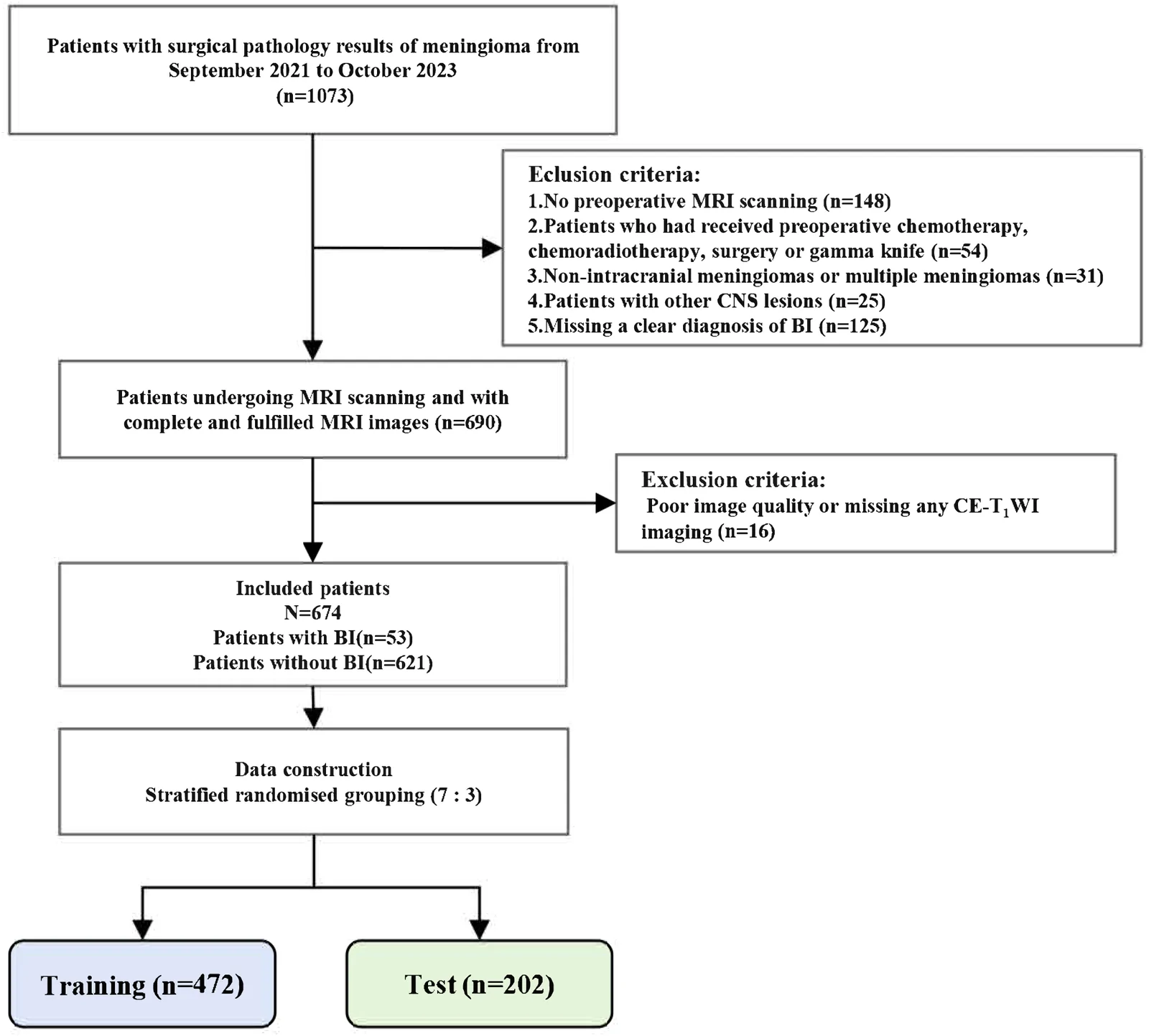
Flowchart of patient screening and cohort allocation. SMOTE was restricted to the training folds during five-fold cross-validation. MRI, magnetic resonance imaging; CE-T_1_WI, contrast-enhanced T_1_-weighted imaging; BI, brain invasion; SMOTE, synthetic minority oversampling technique.

### 2.2 Clinical information and MRI characteristics

The clinical information of the patients included sex and age. The MRI semantic features included tumor location, tumor morphology, tumor margins, tumor enhancement features, dural tailing signs, and peritumor edema. These clinical and MRI semantic features were used to construct the clinical model (ClinM). The patient characteristics are shown in **Table 1**.

**Table 1:** Comparison of clinical and MRI images between patients with non-BI and BI of meningiomas.

Variable [n (%)]	Non-BI (n=621)	BI (n=53)	Statistic	*p-value*
Age[year, x±s]	56.59 ± 11.49	60.00 ± 10.40	-2.086^&^	0.037^*^
Sex			11.948^#^	<0.001^*^
Women [n (%)]	455 (73.27)	27 (50.94)		
Man [n (%)]	166 (26.73)	26 (49.06)		
Location			0.771^#^	0.380
Non-cranial base [n (%)]	384 (61.84)	36 (67.92)		
Base of the skull [n (%)]	237 (38.16)	17 (32.08)		
Tumor morphology			18.381^#^	<0.001^*^
Irregularly [n (%)]	235 (37.84)	36 (67.92)		
Round or oval [n (%)]	386 (62.16)	17 (32.08)		
Tumor margins			14.989^#^	<0.001^*^
Non-smooth [n (%)]	372 (59.90)	46 (86.79)		
Smooth [n (%)]	249 (40.10)	7 (13.21)		
enhancement			20.535^#^	<0.001^*^
Uneven [n (%)]	327 (52.66)	45 (84.91)		
Evenness [n (%)]	294 (47.34)	8 (15.09)		
Dural tail sign			1.471^#^	0.225
No [n (%)]	25 (4.03)	4 (7.55)		
Yes [n (%)]	596 (95.97)	49 (92.45)		
Peritumoral edema			42.871^#^	<0.001^*^
No [n (%)]	326 (52.50)	3 (5.66)		
Yes [n (%)]	295 (47.50)	50 (94.34)		

*P < 0.05 is considered statistically significant difference, ^&^two independent samples t-test, ^#^chi-square test. BI, brain invasion.

### 2.3 Imaging data acquisition

All enrolled patients were scanned using a Skyra, Trio A Tim, or Prisma 3.0T MRI scanner (Siemens Healthcare, Erlangen, Germany) with 20-channel head coils. All scans were performed using the same sequence, with the acquisition parameters listed in **Table 2**.

**Table 2:** The scan parameters of each sequence.

Acquisition parameter	Prisma	Skyra	Trio
Sequences	CE-T_1_WI	CE-T_1_WI	CE-T_1_WI
Direction	TRA	TRA	TRA
TR(ms)	190	240	250
TE(ms)	2.46	2.47	2.46
Slice thickness(mm)	5	5	5
Slice gap(mm)	1.5	1.5	1.5
Slice number	19	21	21
Matrix	216×286	233×320	200×320
FOV(mm^2^)	230×230	240×232.5	220×179.7

CE-T1WI, contrast enhanced T1-weighted imaging; TRA, transverse; TR, repetition time; TE, echo time; FOV, field of view.

### 2.4 Tumor segmentation and image preprocessing

The complete contrast-enhanced T_1_-weighted images (CE-T_1_WI) were preprocessed, involving N4 bias field correction (16), normalization (17), and image resampling to an isotropic voxel size of 1 × 1 × 1 mm³ (18) to reduce intensity non-uniformity, standardize image intensity, and improve feature robustness. The tumor was manually outlined using ITK-SNAP software (version 3.8.0, http://www.itksnap.org) by two radiologists (with 3 and 10 years of experience in neuroimaging) who were blinded to the patients’ clinical conditions. The regions of interest (ROIs) were manually delineated on each slice of the tumor without including the surrounding brain tissue. Another senior radiologist (with 15 years of experience in neuroimaging) reviewed all ROIs. If the difference in the delineated tumor volume was ≥5%, the senior radiologist determined the final tumor boundary to ensure the accuracy of the segmentation.

Twenty scans from each MRI scanner (60 cases in total) were randomly selected for re-segmentation by the two radiologists, and the intraclass correlation coefficients (ICCs) were calculated to assess the robustness and reproducibility of the extracted features. The ICC for repeated ROI delineation was 0.88, indicating good reproducibility. Radiomics features with ICC > 0.80 were considered reproducible and retained for subsequent analysis.

### 2.5 Radiomics and deep transfer learning feature extraction

Radiomics features were extracted from three-dimensional tumor ROIs using the PyRadiomics package (https://pyradiomics.readthedocs.io/en/stable/). For deep transfer learning (DTL) feature extraction, a 2.5D image representation was generated by selecting the maximum cross-sectional slice of each tumor together with the adjacent slices located 1 mm and 2 mm above and below the maximal section. The five selected slices were not combined into a single multi-slice input. Instead, each slice was treated as an independent 2D sample. Because the original MRI slices were single-channel grayscale images, each slice was replicated three times along the channel dimension (R = G = B) to generate a three-channel pseudo-RGB image (3 × H × W), thereby meeting the input requirements of the pretrained Inception-v3 network. Thus, each case generated five independent three-channel pseudo-RGB images, which were separately processed by the pretrained Inception-v3 network for DTL feature extraction. To preserve sufficient peritumoral information, the ROI was expanded by 10 mm beyond the tumor boundary while removing irrelevant contralateral brain tissue. An Inception-v3 network pretrained on the ImageNet (ILSVRC-2012) dataset was used for DTL feature extraction. The final classification layer was replaced with a two-class output layer, while the remaining layers were initialized using the ImageNet-pretrained weights. The entire network was subsequently fine-tuned on the meningioma training data, with all pretrained layers remaining trainable and no layers frozen. Network training was performed using the Adam optimizer with an initial learning rate of 1 × 10^-4^, a batch size of 16, and binary cross-entropy loss for a maximum of 100 epochs. Early stopping was applied by monitoring the validation loss with a patience of 10 epochs. Training was performed using an NVIDIA GeForce RTX 3060 GPU. To improve the interpretability of the fine-tuned Inception-v3 network, gradient-weighted class activation mapping (Grad-CAM) was applied to the final convolutional layer of Inception-v3. The resulting activation maps were used to visualize the image regions contributing to the network predictions. After fine-tuning, a 2048-dimensional feature vector was extracted from the global average pooling layer preceding the final classification layer for each input slice. To reduce the high dimensionality of the DTL features, principal component analysis (PCA) was applied separately to the feature vectors corresponding to each of the five slice positions. For each slice position, the 2048-dimensional feature vector was reduced to 8 principal components. The reduced feature vectors from the five slices were then concatenated at the patient level, resulting in a 40-dimensional DTL feature representation for each patient. These DTL features were subsequently used for model development either independently or in combination with radiomics features.

### 2.6 Model development

The entire cohort was first divided into a training set and an independent test set using stratified sampling at an approximate ratio of 7:3. All feature processing, feature selection, and model development procedures were performed using the training set only, whereas the independent test set was reserved for final model evaluation. Radiomics and DTL features were integrated using an early-fusion strategy, followed by a multistep feature selection procedure. Within the training cohort, five-fold stratified cross-validation was performed for internal model development and evaluation. In each cross-validation iteration, the training and validation portions were first separated The Inception-v3 network was fine-tuned exclusively using the training portion of the corresponding fold. DTL features were then extracted separately from the training and validation portions using the fold-specific fine-tuned network. PCA was fitted only on the DTL features from the training portion and subsequently applied to the corresponding validation portion. All data-driven feature-selection procedures were performed using only the training portion of the corresponding fold. First, features significantly associated with BI were identified using the Mann–Whitney U test, and features with *P* < 0.05 were retained. Subsequently, highly correlated features were removed according to the Spearman correlation coefficient (threshold = 0.90) using a greedy recursive elimination strategy to minimize feature redundancy. The remaining features were further selected using least absolute shrinkage and selection operator (LASSO) regression with 10-fold cross-validation. Features with non-zero coefficients at the optimal regularization parameter (λ) were retained for model construction.

After feature selection in each cross-validation iteration, the Synthetic Minority Over-sampling Technique (SMOTE; k_neighbors = 5) was applied exclusively to the training portion of the corresponding fold to address class imbalance. The validation portion retained its original class distribution and was not involved in SMOTE fitting or synthetic sample generation. The selected features were then used to train an Extra Trees classifier with n_estimators = 60, max_depth = 5, min_samples_split = 2, and random_state = 0. The classifier was fitted on the SMOTE-resampled training fold and evaluated on the corresponding original, non-oversampled validation fold. This procedure was repeated independently across all five folds. The independent test set was not involved in feature selection, oversampling, cross-validation, or model fitting and remained completely untouched until the final model evaluation.

Five predictive models were constructed, including a clinical model (ClinM), radiomics model (RadM), deep transfer learning model (DTLM), deep transfer learning-radiomics model (DTLRM), and a combined clinical-radiomics-DTL model (ComM). A schematic overview of the workflow is presented in **Figure 2**.

**Figure 2:**
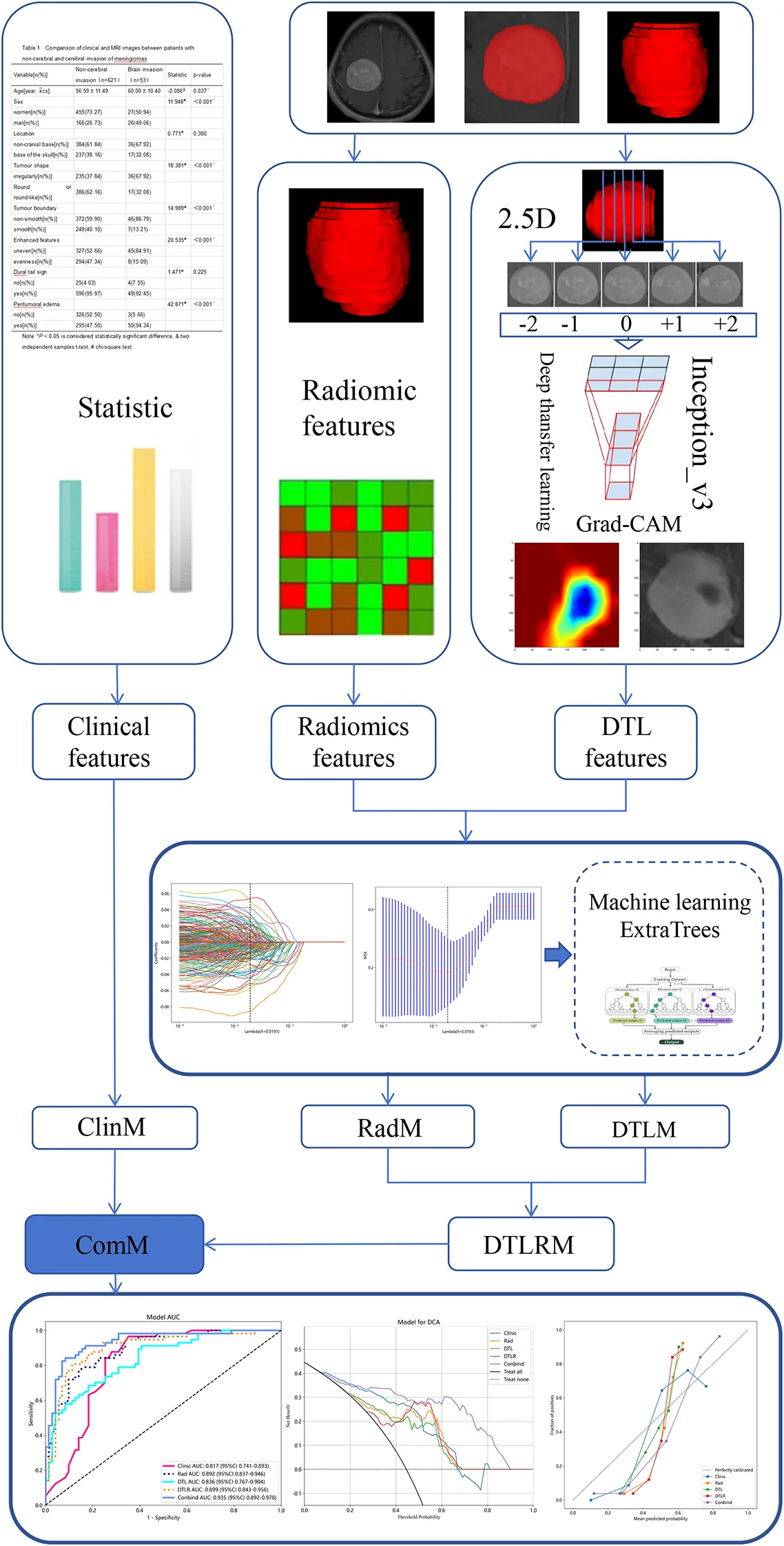
Schematic overview of the study workflow. DTL, deep transfer learning; DTLRM, deep transfer learning-radiomics model.

### 2.7 Statistical analysis

Statistical analysis were performed using SPSS version 20.0, R version 3.6.3, and Python version 3.8.

Continuous variables are presented as the mean ± standard deviation or median (interquartile range), depending on data distribution. Categorical variables are presented as frequencies and percentages. Comparisons between two groups were performed using the independent-samples *t* test or Mann–Whitney *U* test for continuous variables and the chi-square test or Fisher’s exact test for categorical variables.

Model performance was evaluated using receiver operating characteristic (ROC) analysis by calculating the area under the curve (AUC), accuracy (ACC), sensitivity (SEN), specificity (SPE), positive predictive value (PPV), and negative predictive value (NPV). AP/PR-AUC was additionally evaluated to account for class imbalance. Differences in AUCs between models were assessed using the DeLong test. Model calibration was evaluated using the Hosmer–Lemeshow goodness-of-fit test and calibration curves, and the Brier score. Decision curve analysis (DCA) was performed to assess the clinical utility of each model by estimating the net benefit across different threshold probabilities. A two-sided *P* value < 0.05 was considered statistically significant.

## 3 Results

### 3.1 General clinical data and MRI characteristics

The demographic and baseline characteristics of the study patients are summarized in **Table 1**. A total of 674 patients with meningioma were enrolled, including 53 BI patients and 621 non-BI patients. Independent-samples t tests and chi-square tests were used to compare the baseline demographic and MRI characteristics of meningioma patients with and without BI, and the results revealed statistically significant differences in age, sex, tumor morphology, tumor enhancement features, tumor margins, and peritumor edema between the groups (*P* < 0.05). The details are shown in **Table 1**.

### 3.2 Feature screening

A total of 1834 radiomics features, including first-order features, shape-based features, and texture features, were extracted from CE-T1WI for each patient. The extracted radiomics features included first-order, shape-based, gray level co-occurrence matrix (GLCM), gray level dependence matrix (GLDM), gray level run length matrix (GLRLM), gray level size zone matrix (GLSZM), and neighboring gray tone difference matrix (NGTDM) features.

The five 2.5D tumor slices were independently processed for DTL feature extraction. A 2,048-dimensional DTL feature vector was extracted from each input slice. PCA was subsequently applied to the DTL features corresponding to each slice position, reducing the dimensionality from 2,048 to 8 components per slice. The five reduced feature vectors were then concatenated, resulting in a 40-dimensional patient-level DTL feature representation. After feature-level fusion, a total of 1,874 radiomics and DTL features were obtained. After early fusion of the 40 PCA-reduced DTL features with the 1,834 radiomics features, a total of 1,874 candidate DTL-radiomics features were obtained for subsequent feature selection. To improve the interpretability of the DTL model, Grad-CAM was applied to the final convolutional layer of Inception-v3 to visualize the image regions contributing to the network predictions (**Figure 3**).

**Figure 3:**
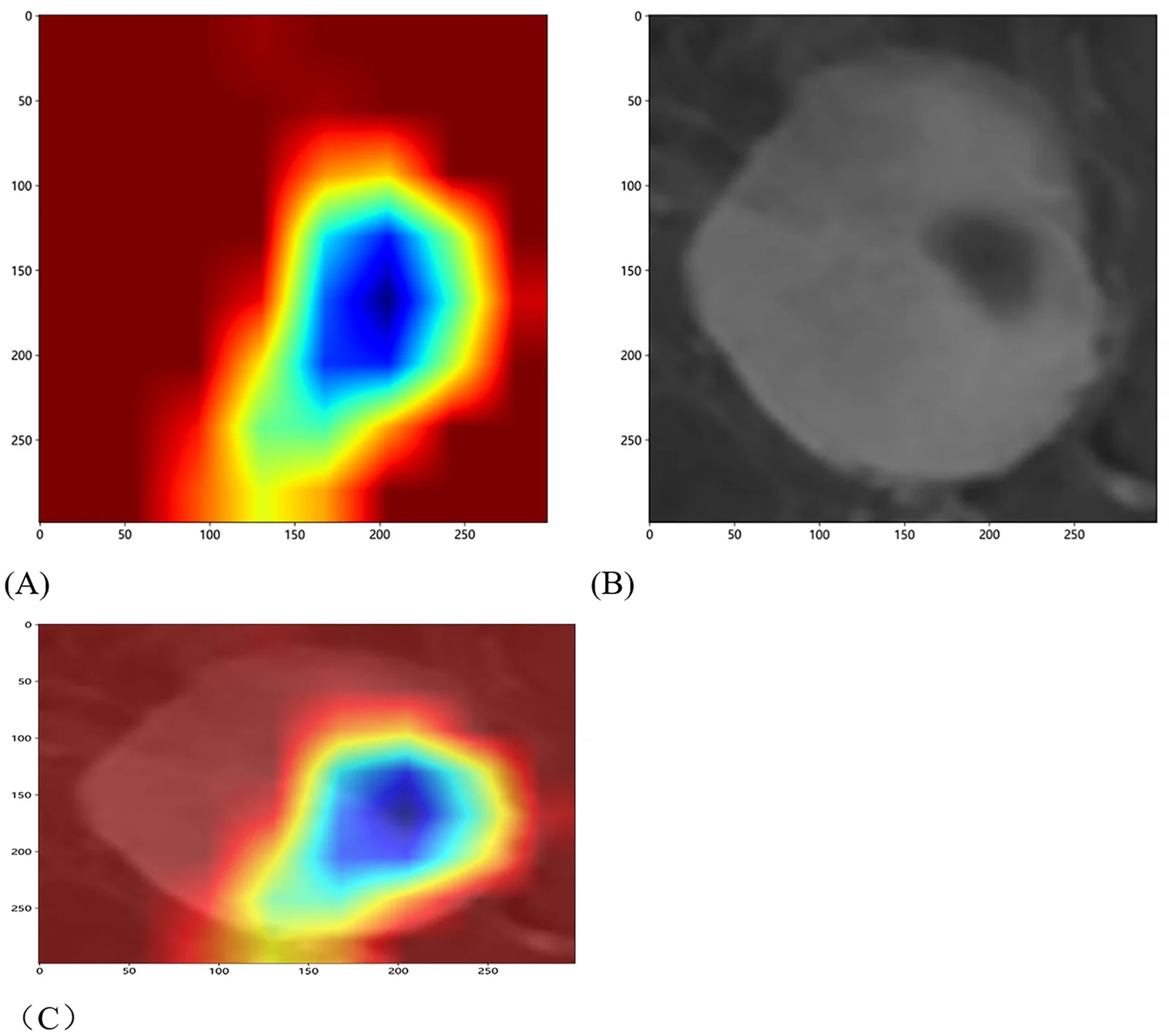
Grad-CAM visualization of the DTL model. **(A)** DTL feature activation map. **(B)** Original image of the maximum tumor cross-sectional slice. **(C)** Grad-CAM activation map overlaid on the original image. Grad-CAM, gradient-weighted class activation mapping; DTL, deep transfer learning.

Following the multistep feature-selection procedure described in the Methods section, LASSO regression ultimately retained 137 features with non-zero coefficients, including 125 radiomics features and 12 DTL features. These features were collectively referred to as the selected DTL-radiomics features. Detailed results of the radiomics feature-selection process, including the number and percentage of features retained at each step, are provided in **Supplementary Table S2**. The LASSO coefficient profiles and the corresponding 10-fold cross-validation results for selection of the optimal regularization parameter (λ) are shown in **Figures 4A, B**, respectively.

**Figure 4:**
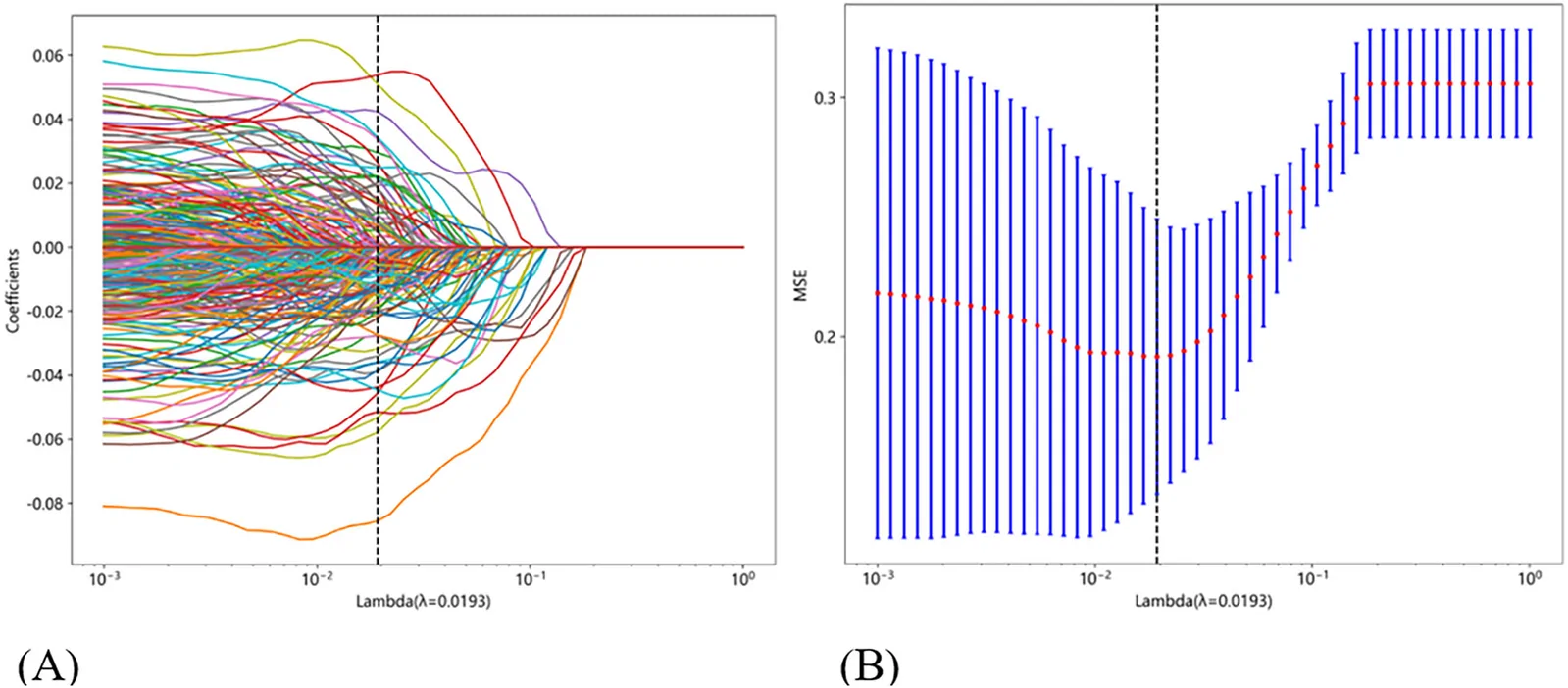
LASSO feature selection of DTL-radiomics features. **(A)** LASSO coefficient profiles of the candidate features. **(B)** Ten-fold cross-validation curve for selection of the optimal regularization parameter (λ). DTL, deep transfer learning; LASSO, least absolute shrinkage and selection operator.

### 3.3 Model construction and evaluation

A summary of the prediction performance metrics of the five models in the training and test sets is shown in **Table 3** and **Figure 5**. The AUC values of each model were greater than 0.800 in both the training and test sets. ComM outperformed the other models in both the training and test sets (training set: ComM: 0.977 vs. ClinM: 0.886, RadM: 0.944, DTLM: 0.857, DTLRM: 0.955; test set: ComM: 0.935 vs. ClinM: 0.817, RadM 0.892, DTLM: 0.836, DTLRM: 0.899). ComM exhibited the best performance for predicting brain invasion in meningiomas. ComM also achieved the highest AP/PR-AUC in the training and test sets (0.968 and 0.916, respectively; **Supplementary Table S4**). Detailed 95% confidence intervals for the performance metrics are provided in **Supplementary Table S1**.

**Table 3:** Predictive effectiveness of all models in the training and test sets.

Set	Model	ACC	AUC	95% CI	SEN	SPE	PPV	NPV
Training set	ClinM	0.811	0.886	0.848 - 0.923	0.859	0.766	0.772	0.855
	RadM	0.892	0.944	0.918 - 0.969	0.901	0.883	0.877	0.907
	DTLM	0.780	0.857	0.816 - 0.899	0.866	0.701	0.728	0.850
	DTLRM	0.885	0.955	0.934 - 0.976	0.845	0.922	0.909	0.866
	ComM	0.926	0.977	0.964 - 0.990	0.915	0.935	0.929	0.923
Test set	ClinM	0.773	0.817	0.741 - 0.893	0.877	0.690	0.694	0.875
	RadM	0.812	0.892	0.837 - 0.946	0.754	0.859	0.811	0.813
	DTLM	0.727	0.836	0.767 - 0.904	0.895	0.592	0.637	0.875
	DTLRM	0.844	0.899	0.843 - 0.956	0.789	0.887	0.849	0.840
	ComM	0.875	0.935	0.892 - 0.978	0.825	0.915	0.887	0.867

ClinM, clinical model; RadM, radiomic model; DTLM, deep transfer learning model; DTLRM, deep transfer learning radiomics model; ComM, combined model.

**Figure 5:**
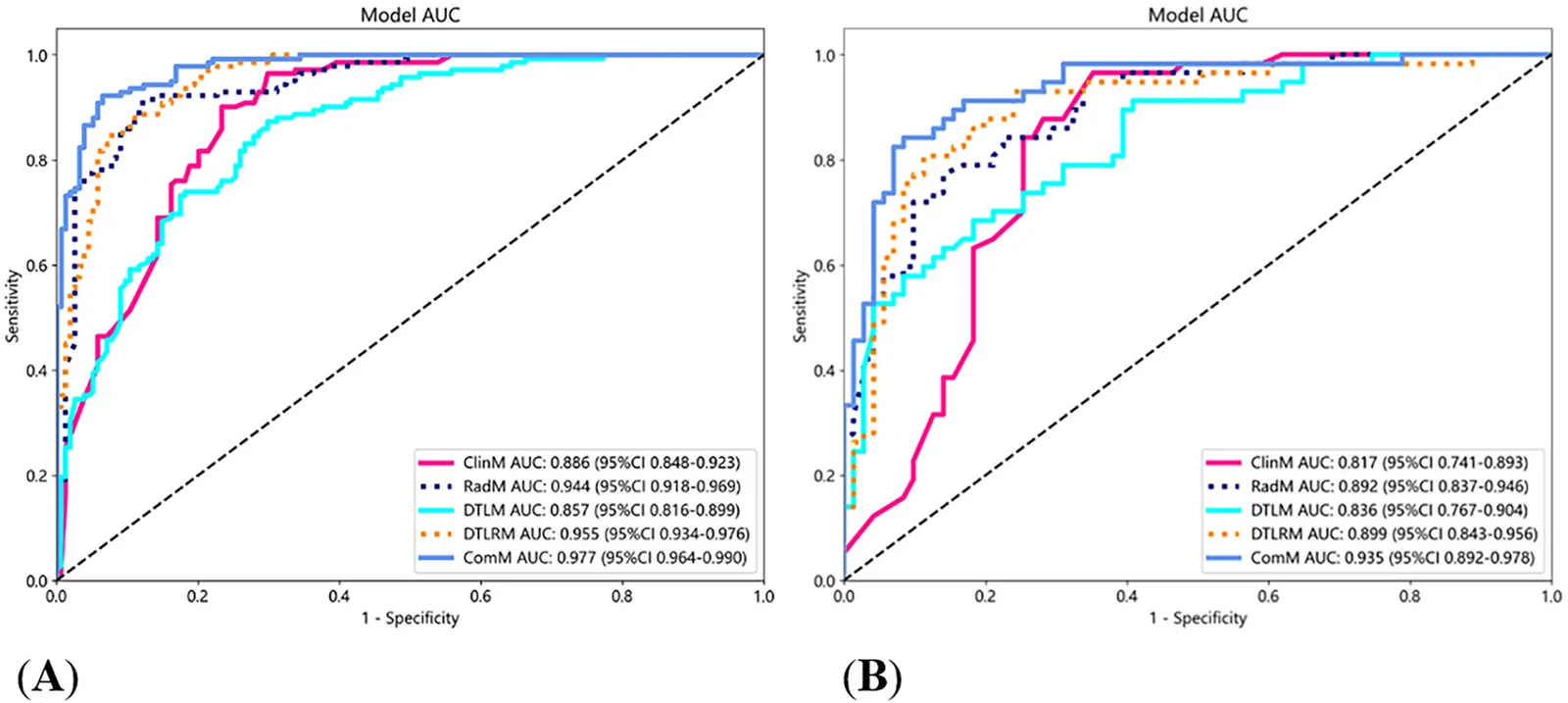
Receiver operating characteristic (ROC) curves of ClinM, RadM, DTLM, DTLRM, and ComM in the **(A)** training and **(B)** test sets. ClinM, clinical model; RadM, radiomic model; DTLM, deep transfer learning model; DTLRM, deep transfer learning radiomics model; ComM, combined model.

### 3.4 Model comparison

The Delong test was used to perform pairwise comparisons of the AUC values of each model. The *P* values for ComM compared with ClinM, RadM, DTLM, and DTLRM in the test set were all less than 0.05, and the difference was statistically significant, as shown in **Figure 6**. As shown in **Table 4**, the Hosmer–Lemeshow goodness-of-fit test indicated good fit for each model. The ComM also achieved the lowest Brier scores in both the training and test sets (0.073 and 0.113, respectively), with detailed results for all models provided in **Supplementary Table S3**. The calibration curves revealed that ComM was closest to the ideal curve in both the training and test sets, as shown in **Figure 7**, indicating that the model predicted meningioma BI with better calibration than the other models did. The clinical net gain of each model was evaluated by DCA, and as shown in **Figure 8**, ComM had the highest net gain over a wide range of threshold probabilities relative to ClinM, RadM, DTLM, and DTLRM, indicating that ComM had a better clinical net gain in the preoperative prediction of meningioma BI.

**Figure 6:**
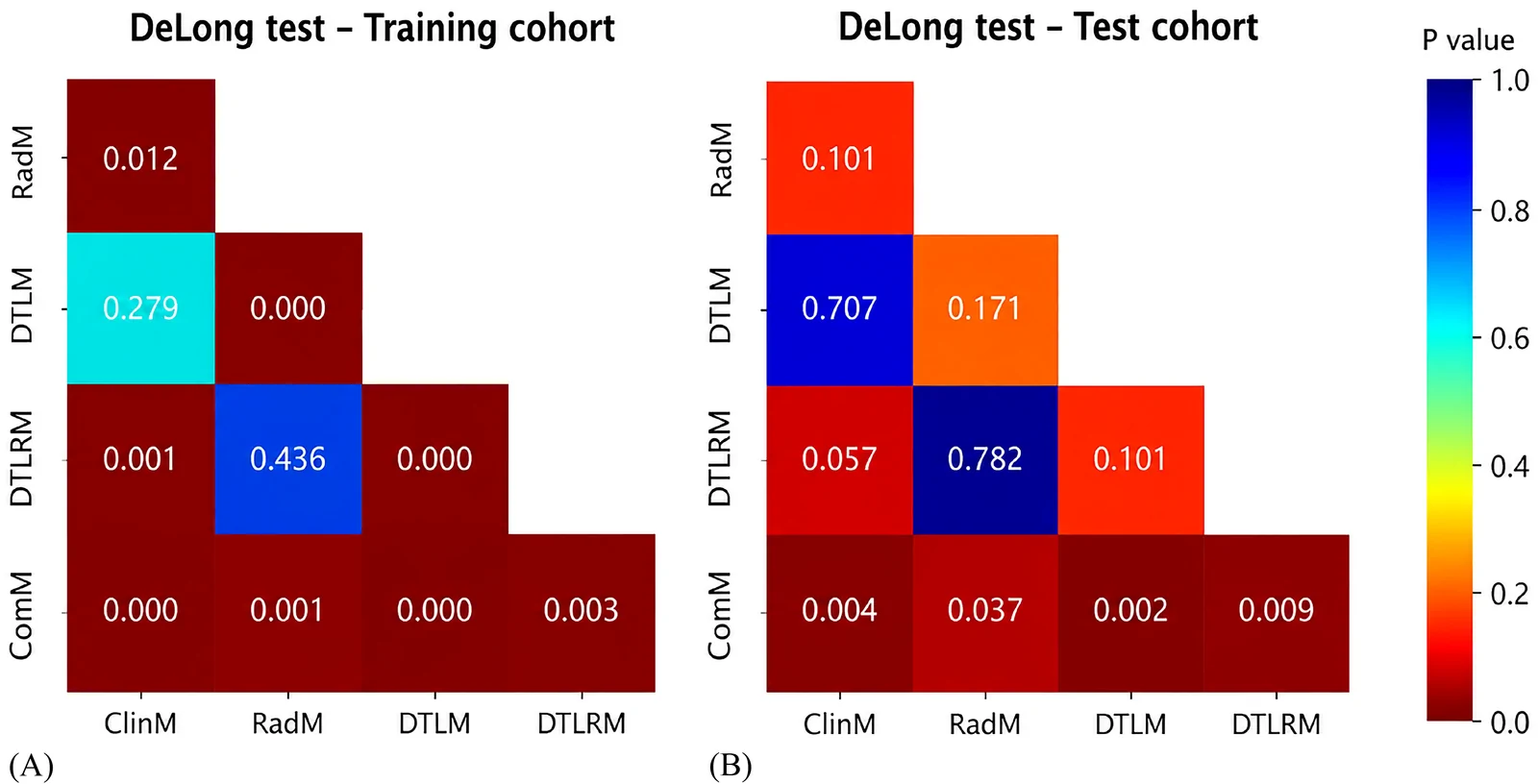
Pairwise *DeLong test* comparisons of AUCs among the five models in the **(A)** training and **(B)** test sets. ClinM, clinical model; RadM, radiomic model; DTLM, deep transfer learning model; DTLRM, deep transfer learning radiomics model; ComM, combined model.

**Table 4:** Hosmer-lemeshow tests.

Set	ClinM	RadM	DTLM	DTLRM	ComM
Train	0.984	0.044	0.164	0.071	0.999
Test	0.746	0.108	0.338	0.082	0.818

**Figure 7:**
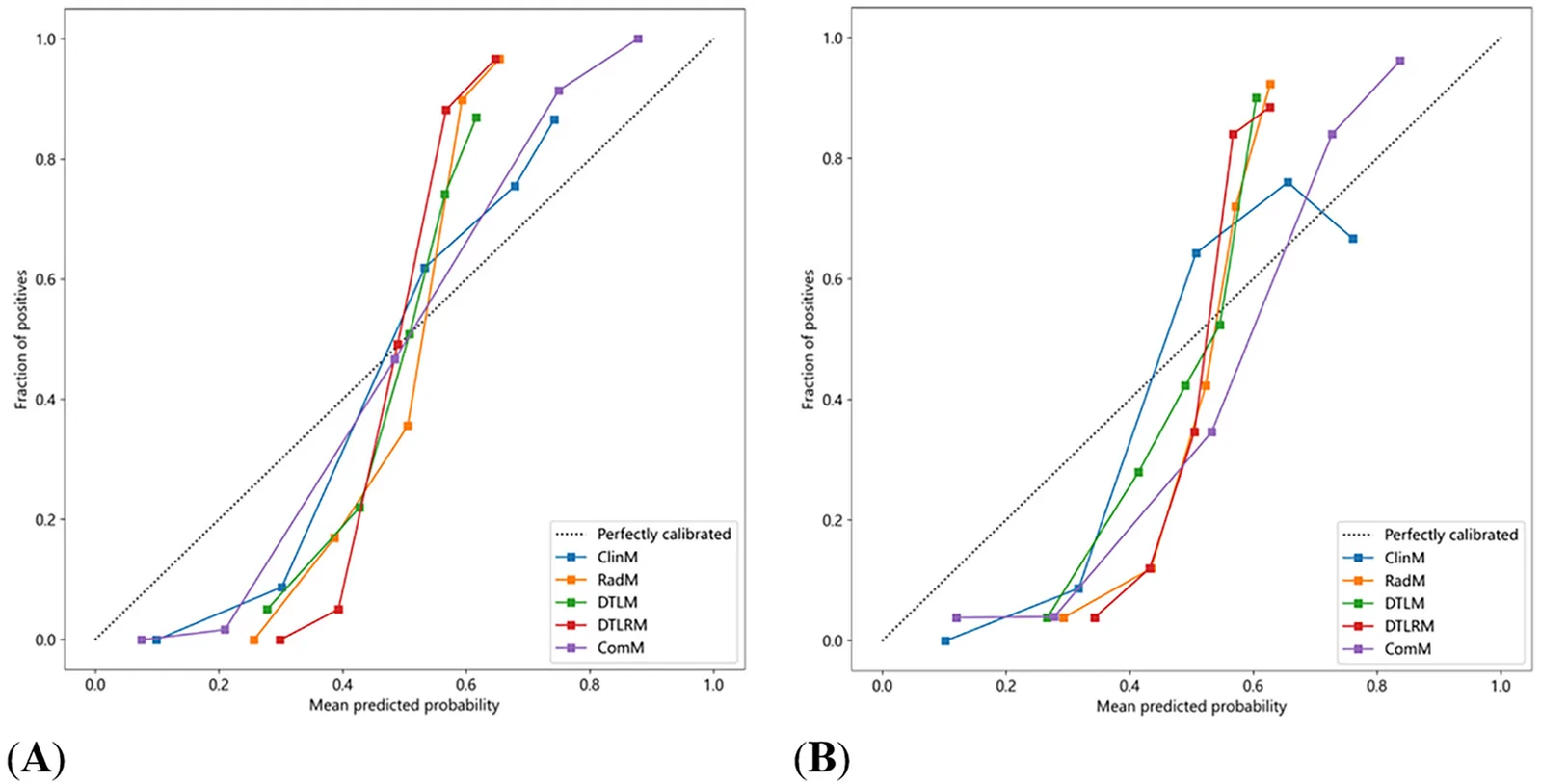
Calibration curves of ClinM, RadM, DTLM, DTLRM, and ComM in the **(A)** training and **(B)** test sets. ClinM, clinical model; RadM, radiomic model; DTLM, deep transfer learning model; DTLRM, deep transfer learning radiomics model; ComM,combined model.

**Figure 8:**
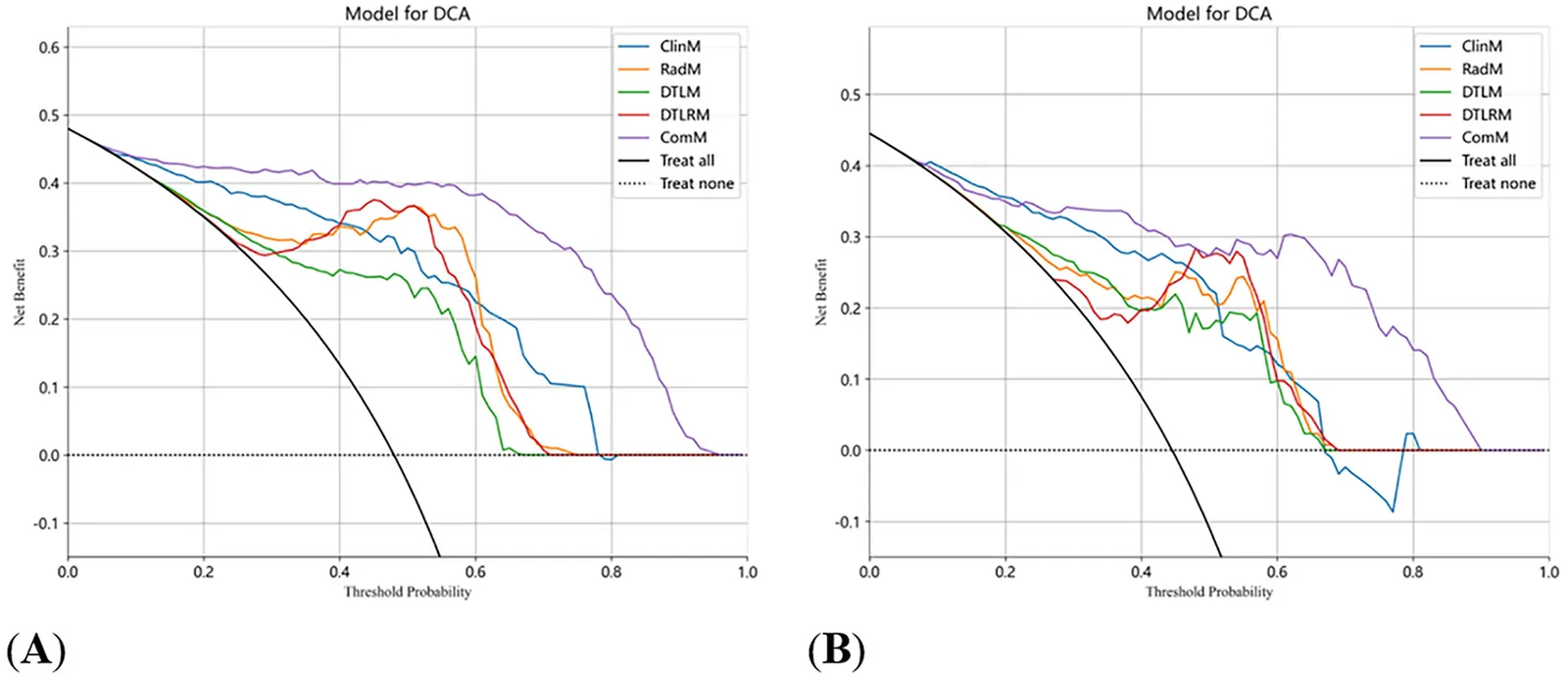
Decision curve analysis (DCA) for ClinM, RadM, DTLM, DTLRM, ComM in the training and test sets; **(A)** training set, **(B)** test set. ClinM, clinical model; RadM, radiomic model; DTLM, deep transfer learning model; DTLRM, deep transfer learning radiomics model; ComM, combined model.

## 4 Discussion

Using the clinical information of meningioma patients and tumor information from 2.5D images, ComM, ClinM, RadM, DTLM, and DTLRM were constructed. Among them, ComM showed the best performance, with an AUC of 0.977 in the training set and 0.935 in the test set, suggesting that this model could be an efficient method to predict the BI of meningioma preoperatively.

In this study, we developed and validated various prediction models based on 2.5D image features extracted from preoperative CE-T1WI using DL and clinical features. Previous studies (19) have used mostly 2D or 3D imaging data for diagnosis; traditional 2D models have certain limitations in capturing overall tumor features, and they provide less information than 3D models do. Therefore, 3D models usually perform better. However, 3D models require more complex model structures and more training data and model parameters, which are prone to overfitting problems. In this study, the 2.5D images used for DTL feature extraction included the maximum tumor slice and the slices located 1 mm and 2 mm above and below it, providing more spatial information than traditional 2D imaging while requiring fewer computational resources than 3D imaging. The DTL features were extracted using a convolutional neural network pretrained via transfer learning, and the extracted features were more comprehensive, providing more tumor information and improving the model’s prediction performance. This additional information not only helps doctors better understand the prediction results of the model but also improves the accuracy of diagnosis, thereby providing more precise guidance for patient treatment.

DL is a computational model that learns data representations with multiple levels of abstraction by using multiple processing layers. DL discovers the complex structures of big data through the backpropagation algorithm, which instructs the machine to modify its internal parameters (20). DL requires a large dataset for training and increased computing resources. To address problems such as insufficient data volume and poor computer performance, the transfer learning strategy, which applies the knowledge learned in one field to another field, was used in this study. This approach enables the model to utilize the training results of the existing dataset to improve its efficiency for solving new tasks. Zeng et al. (21) used transfer learning to extract DL features for early gastric cancer lymph node metastasis prediction and achieved good model prediction performance, with AUCs of 0.901 and 0.915 in the internal validation and external validation cohorts, respectively. Zhu et al. (22) achieved good performance on the training set and test set for preoperative meningioma grading using DTL, with AUCs of 0.891 and 0.811; the results of these studies are consistent with those of our study, suggesting that transfer learning performs well in preoperative prediction.

RadM, which was built using radiomics features, performed well in both the training set and test set. The Hosmer–Lemeshow test was used to evaluate calibration, and the P value of RadM in the training set was 0.044, suggesting that the poor fit of this model in the training set may be due to the poor fitting of radiomics features by the Extra Trees algorithm. In future studies, we will attempt to use other machine learning methods to improve the fit of RadM. ComM with DTL features had the highest predictive power and good model fit.

The AUC of DTLM was 0.857 [95% confidence interval (CI): 0.816, 0.899] in the training set and 0.836 [95% CI: 0.767, 0.904] in the test set. The AUCs of DTLRM, which combined radiomics features and DTL features, were 0.955 and 0.899 in the training set and test set, respectively, and DTLRM outperformed RadM and DTLM [DTLRM: 0.899 vs. RadM: 0.892, DTLM: 0.836]. This study revealed that the addition of DTL features improved model prediction performance and effectively reflected intratumor heterogeneity and predicted BI of meningiomas. The DTL and radiomics features capture the corresponding features of the tumor images. Both radiomics features and DTL features are derived from quantitative measurements of tumors and may play complementary roles. In this study, the AUCs of the radiomics feature model and DTL feature model in the test set were greater than 0.8, and the radiomics feature model was slightly better than the DTL feature model (RadM: 0.892, DTLM: 0.836), which may be because radiomics features are extracted from the entire 3D tumor volume, whereas DTL features in this study were derived from selected 2.5D tumor slices. This model also reflects the difference between radiomics features and DTL features in quantifying the information of tumor heterogeneity (18). Yang et al. (23) graded meningiomas using a deep learning radiomics model (DLRM) trained on enhanced T1-weighted images (T1WI), which outperformed both the radiomics and DL models [training set AUCs: 0.988 vs. 0.980 vs. 0.892; test set AUCs: 0.935 vs. 0.918 vs. 0.718]. The DLRM had good personalized non-invasive prediction ability, and its clinical application value is better than that of the radiomics model. Zeng et al. (21) integrated clinical variables with multimodel data and combined radiomics features with DL features to distinguish lymph node metastasis status in early gastric cancer with good predictive ability, which provided information for selecting individualized treatment options. Zheng et al. (24) constructed a DL radiomics nomogram based on augmented CT for predicting the histological differentiation grades of squamous cell carcinoma of the head and neck, and nomogram showed good predictive results. The results of these studies are in general agreement with those of the present study. Grad-CAM visualization provided additional qualitative insight into the image regions contributing to the DTL model predictions. Visual inspection of the activation maps suggested that the network attended to tumor regions and their surrounding interfaces. Such attention patterns may be relevant to imaging characteristics associated with brain invasion, including irregular tumor margins, alterations at the tumor–brain interface, and peritumoral edema. These observations suggest that the DTL model may capture imaging information related to tumor invasiveness; however, they should be interpreted cautiously because the Grad-CAM analysis in the present study was qualitative. No quantitative assessment of activation-map localization was performed. Further studies incorporating quantitative explainability metrics and direct radiological or pathological correlation are warranted to validate the biological significance of these activation patterns. In this study, Extra Trees was selected because it can model nonlinear relationships in high-dimensional clinical, radiomics, and DTL feature spaces, while its additional randomization may help reduce overfitting. Compared with boosting-based methods such as XGBoost, CatBoost, and LightGBM, it also provides a relatively simple ensemble framework with less dependence on extensive sequential optimization. The results of this study revealed that the efficacy of DTLRM and ComM, which included DTL features, was better than that of ClinM and RadM, suggesting that DTL can provide other features related to the biological behavior of meningioma BI that are different from radiomics features. From a clinical perspective, preoperative estimation of BI risk may provide complementary information for surgical planning. In patients predicted to have a high risk of BI, surgeons may anticipate a more adherent or infiltrative tumor–brain interface and prepare for a potentially more complex resection. Conversely, a low predicted risk of BI may help avoid unnecessarily aggressive surgical strategies when supported by other clinical and imaging findings. Thus, the proposed model may serve as an adjunctive preoperative risk-stratification tool rather than a replacement for clinical judgment. However, whether model-assisted risk assessment can improve surgical decision making or patient outcomes requires further evaluation in prospective multicenter studies.

## 5 Limitations

First, this study was retrospective and may have selection bias. Second, the sample size of meningioma patients with BI in this study was relatively small, and there was a significant difference in the sample size between the two groups. Therefore, SMOTE was applied to address class imbalance during model development.

Finally, this was a single-center retrospective study without external validation. Although an independent test set was used for internal performance assessment, all MRI examinations were performed using Siemens 3.0T scanners. Therefore, the robustness and generalizability of the proposed models across different scanner vendors, field strengths, and acquisition protocols remain uncertain. External validation using larger, multicenter, and more heterogeneous cohorts incorporating different MRI platforms and acquisition settings is required before clinical implementation.

## 5 Conclusion

ClinM, RadM, DTLM, DTLRM, and ComM constructed based on CE-T_1_WI all had good efficacy in predicting the BI of meningiomas (AUC > 0.8 for all). DTLRM and ComM, which included DTL features, outperformed ClinM and RadM, suggesting that DTL can provide additional features that are related to the biological behavior of meningioma BI and different from radiomics features. ComM had the best efficacy in predicting meningioma BI. These findings suggest the potential clinical utility of the combined model for preoperative prediction of meningioma BI; however, further external validation is required before clinical implementation.

## Data Availability

The raw data supporting the conclusions of this article will be made available by the authors, without undue reservation.
