# Supplementary material for: Non-invasive preoperative MRI-based deep transfer learning radiomics model for predicting meningioma brain invasion
Source: Front Oncol. 2026 Oct 7;16:1935311. doi: 10.3389/fonc.2026.1935311 (PMC13683121; doi:10.3389/fonc.2026.1935311)
Supplement: Supplementary file 1 [file Table1.docx]

**Supplement Materials**

**Non-Invasive Preoperative MRI-Based Deep Transfer Learning Radiomics Model for Predicting Meningioma Brain Invasion**

**Supplementary Table 1** Diagnostic performance of different models in the training and test sets.

**Supplementary Table S2.** Number and percentage of radiomics features retained after each feature-selection step.

**Supplementary Table S3.** AUC and Brier scores of the five predictive models in the training and test sets.

**Supplementary Table S4.** AP/PR-AUC values of the five predictive models in the training and test sets.

**Supplementary Table 1** Diagnostic performance of different models in the training and test sets.

| **Set** | **Model** | **ACC (95%CI)** | **AUC (95%CI)** | **SEN (95%CI)** | **SPE (95%CI)** | **PPV (95%CI)** | **NPV (95%CI)** |
| --- | --- | --- | --- | --- | --- | --- | --- |
| Training  set | ClinM | 0.811 (0.762-0.854) | 0.886 (0.848-0.924) | 0.859 (0.791-0.906) | 0.766 (0.694-0.826) | 0.772 (0.701-0.831) | 0.855 (0.787-0.904) |
|  | RadM | 0.892 (0.853-0.924) | 0.944 (0.918-0.969) | 0.901 (0.841-0.941) | 0.883 (0.822-0.924) | 0.877 (0.814-0.921) | 0.907 (0.849-0.944) |
|  | DTLM | 0.780 (0.728-0.825) | 0.857 (0.816-0.899) | 0.866 (0.800-0.911) | 0.701 (0.624-0.769) | 0.728 (0.656-0.789) | 0.850 (0.778-0.902) |
|  | DTLRM | 0.885 (0.845-0.918) | 0.955 (0.934-0.976) | 0.845 (0.776-0.895) | 0.922 (0.867-0.956) | 0.909 (0.848-0.947) | 0.866 (0.805-0.910) |
|  | ComM | 0.926 (0.893-0.952) | 0.977 (0.964-0.990) | 0.915 (0.857-0.953) | 0.935 (0.884-0.967) | 0.929 (0.874-0.961) | 0.923 (0.870-0.955) |
| Test  set | ClinM | 0.773 (0.692-0.839) | 0.817 (0.741-0.893) | 0.877 (0.764-0.938) | 0.690 (0.575-0.786) | 0.694 (0.580-0.789) | 0.875 (0.764-0.938) |
|  | RadM | 0.812 (0.734-0.873) | 0.892 (0.837-0.946) | 0.754 (0.624-0.854) | 0.859 (0.755-0.923) | 0.811 (0.686-0.894) | 0.813 (0.711-0.885) |
|  | DTLM | 0.727 (0.642-0.801) | 0.836 (0.767-0.904) | 0.895 (0.788-0.953) | 0.592 (0.475-0.701) | 0.637 (0.528-0.734) | 0.875 (0.753-0.941) |
|  | DTLRM | 0.844 (0.770-0.901) | 0.899 (0.843-0.956) | 0.789 (0.660-0.883) | 0.887 (0.788-0.946) | 0.849 (0.729-0.921) | 0.840 (0.741-0.906) |
|  | ComM | 0.875 (0.806-0.925) | 0.935 (0.892-0.978) | 0.825 (0.701-0.906) | 0.915 (0.823-0.961) | 0.887 (0.774-0.947) | 0.867 (0.772-0.926) |

**Note:** ACC, accuracy; AUC, area under the receiver operating characteristic curve; SEN, sensitivity; SPE, specificity; PPV, positive predictive value; NPV, negative predictive value; CI, confidence interval. Data are presented as estimates with 95% confidence intervals. The 95% CIs for PPV and NPV were calculated using the Wilson score method.

**Supplementary Table S2.** Number and percentage of radiomics features retained after each feature-selection step.

| **Feature-selection step** | **ICC / selection criterion** | **No. retained** | **Retained (%)** |
| --- | --- | --- | --- |
| Initial extraction | — | 1,834 | 100.0% |
| ICC reproducibility filtering | ICC > 0.80 | 1,562 | 85.2% |
| Mann–Whitney U test | P < 0.05 | 742 | 40.5% |
| Spearman correlation filtering | \|r\| < 0.90 | 286 | 15.6% |
| LASSO | Non-zero coefficients | 125 | 6.8% |

**Note:** A total of 1,834 radiomics features were initially extracted. Radiomics features with ICC > 0.80 were considered reproducible and retained for subsequent feature selection. ICC, intraclass correlation coefficient; LASSO, least absolute shrinkage and selection operator.

**Supplementary Table S3.** AUC and Brier scores of the five predictive models in the training and test sets.

| **Set** | **Model** | **Hosmer–Lemeshow P value** | **Brier score** |
| --- | --- | --- | --- |
| Training  set | ClinM | 0.984 | 0.145 |
|  | RadM | 0.044 | 0.108 |
|  | DTLM | 0.164 | 0.164 |
|  | DTLRM | 0.071 | 0.099 |
|  | ComM | 0.999 | 0.073 |
| Test  set | ClinM | 0.746 | 0.179 |
|  | RadM | 0.108 | 0.143 |
|  | DTLM | 0.338 | 0.191 |
|  | DTLRM | 0.082 | 0.136 |
|  | ComM | 0.818 | 0.113 |

**Note:** Lower Brier scores indicate better overall probabilistic prediction performance. H–L, Hosmer–Lemeshow; ClinM, clinical model; RadM, radiomics model; DTLM, deep transfer learning model; DTLRM, deep transfer learning-radiomics model; ComM, combined model.

**Supplementary Table S4.** AP/PR-AUC values of the five predictive models in the training and test sets.

| **Set** | **Model** | **AP/PR-AUC** |
| --- | --- | --- |
| Training set | ClinM | 0.861 |
|  | RadM | 0.931 |
|  | DTLM | 0.824 |
|  | DTLRM | 0.943 |
|  | ComM | 0.968 |
| Test set | ClinM | 0.774 |
|  | RadM | 0.872 |
|  | DTLM | 0.793 |
|  | DTLRM | 0.879 |
|  | ComM | 0.916 |
